# Differential Effects of Ang-2/VEGF-A Inhibiting Antibodies in Combination with Radio- or Chemotherapy in Glioma

**DOI:** 10.3390/cancers11030314

**Published:** 2019-03-06

**Authors:** Gergely Solecki, Matthias Osswald, Daniel Weber, Malte Glock, Miriam Ratliff, Hans-Joachim Müller, Oliver Krieter, Yvonne Kienast, Wolfgang Wick, Frank Winkler

**Affiliations:** 1 Neurology Clinic and Neurooncology Program at the National Center for Tumor Disease, University Hospital Heidelberg, Im Neuenheimer Feld 400, 69120 Heidelberg, Germany; gergely.solecki@web.de (G.S.); matthias.osswald@med.uni-heidelberg.de (M.O.); Miriam.Ratliff@umm.de (M.R.); wolfgang.wick@med.uni-heidelberg.de (W.W.); 2 German Cancer Consortium (DKTK), Clinical Cooperation Unit Neurooncology, German Cancer Research Center (DKFZ), 69120 Heidelberg, Germany; daniel.weber97@gmx.de (D.W.); malte.glock@web.de (M.G.); 3 Business Unit Service and Customer Care, Carl Zeiss Microscopy GmbH, 07745 Jena, Germany; 4 Neurosurgery Department, University Medical Center Mannheim, 68167 Mannheim, Germany; 5 Pharmaceutical Research and Early Development (pRED), Roche Innovation Center Munich, 82377 Munich, Germany; hans-joachim.mueller@mnet-mail.de (H.-J.M.); Oliver.Krieter@roche.com (O.K.); yvonne.kienast@roche.com (Y.K.)

**Keywords:** Ang-2, antiangiogenic therapy, in vivo imaging, radio- and chemotherapy, VEGF-A

## Abstract

Antiangiogenic strategies have not shown striking antitumor activities in the majority of glioma patients so far. It is unclear which antiangiogenic combination regimen with standard therapy is most effective. Therefore, we compared anti-VEGF-A, anti-Ang2, and bispecific anti-Ang-2/VEGF-A antibody treatments, alone and in combination with radio- or temozolomide (TMZ) chemotherapy, in a malignant glioma model using multiparameter two-photon in vivo microscopy in mice. We demonstrate that anti-Ang-2/VEGF-A lead to the strongest vascular changes, including vascular normalization, both as monotherapy and when combined with chemotherapy. The latter was accompanied by the most effective chemotherapy-induced death of cancer cells and diminished tumor growth. This was most probably due to a better tumor distribution of the drug, decreased tumor cell motility, and decreased formation of resistance-associated tumor microtubes. Remarkably, all these parameters where reverted when radiotherapy was chosen as combination partner for anti-Ang-2/VEGF-A. In contrast, the best combination partner for radiotherapy was anti-VEGF-A. In conclusion, while TMZ chemotherapy benefits most from combination with anti-Ang-2/VEGF-A, radiotherapy does from anti-VEGF-A. The findings imply that uninformed combination regimens of antiangiogenic and cytotoxic therapies should be avoided.

## 1. Introduction

Glioblastoma (GB) is the most common and most malignant adult primary brain tumor [1]. It is associated with a poor prognosis and a high burden for the patient. The standard treatment is maximum safe resection, followed by radiotherapy and concomitant and adjuvant temozolomide (TMZ) chemotherapy. Despite this intensive treatment, overall survival (OS) remains under two years [2], largely because of inherent tumor resistance mechanisms [3,4,5]. Therefore, better therapeutic strategies are urgently needed, which includes those that make standard radio- and chemotherapy more efficient.

GBs are characterized by dense but structurally and functionally abnormal blood vessels, which are driven by a high level of proangiogenic factors, particularly VEGF-A [6,7,8] and Angiopoietin 2 (Ang-2) [9,10,11,12]. Ang-2 inhibition has previously been described to increase the effectiveness of anti-VEGF-A therapy in glioma [9,10,11,13].

The aberrant glioma blood supply is likely to compromise the effects of radio- and chemotherapy in malignant gliomas: due to high levels of tumor hypoxia [6] and potentially also by reduced delivery of TMZ to the glioma cells [14]. Thus, reestablishment of a more physiological microvascular function by antiangiogenic therapies, called vascular normalization, might increase the effectiveness of radio- and/or chemotherapy in gliomas [6,15,16,17,18]. This concept is supported from clinical data outside the brain, where combination of anti-VEGF-A therapies with chemotherapy showed the best antitumor effectivities [19,20]. However, in two phase III clinical trials in frontline GB (AVAglio and RTOG 0825) where standard radiochemotherapy was combined with the anti-VEGF-A antibody bevacizumab (Avastin^®^, Genentech Inc., South San Francisco, CA, USA), progression free survival (PFS), was improved by 4.4 months, while OS was unchanged [21,22]. Similar results were obtained in the EORTC 26101 study where bevacizumab was combined with lomustine chemotherapy vs. lomustine alone in patients with progressive GB [23]. Together these results unequivocally confirm that bevacizumab activity in controlled clinical trials remains far below expectations in newly diagnosed and relapsed GB.

The reason for that is not clear. Next to the possibility that only subgroups of GB patients benefit [4,24,25], other explanations for the so far disappointing overall benefits of anti-VEGF-A therapy include: (1) suboptimal vascular normalization of single VEGF-A inhibition, which is insufficient to increase the effectiveness of cytotoxic therapy and (2) lack of synergy or even detrimental effects for the combination with cytotoxic therapy. For chemotherapy, for example, vascular normalization with partial re-erection of the blood-brain barrier might compromise tumor penetration of the drug.

Therefore, to increase the benefit from antiangiogenic treatment strategies in glioma, it appears necessary to test these two possibilities: by directly comparing how inhibitors of VEGF-A, Ang-2, and both affect multiple critical parameters of tumor biology, and, most importantly, whether that benefits concomitant chemotherapy, radiotherapy, or both. Therefore, in this study we provide such a comprehensive comparison, making use of a newly developed multi-parameter longitudinal in vivo multi-photon microscopy technology. The results speak for a complex and dynamic system of interactions between the treatment modalities, and finally suggest that anti-Ang-2/VEGF-A is the best combination partner for chemotherapy, but anti-VEGF-A for radiotherapy.

## 2. Results

### 2.1. A Dynamic Multi-Parameter Microscopy Model to Study Therapeutic Interactions

To achieve parallel inhibition of both human and mouse VEGF-A and Ang-2, a bispecific antibody was used that employed the CrossMab technology [10,11,26,27,28] (the humanized antibody is vanucizumab [29,30]), combining the anti-Ang-2 specific IgG1 antibody LC06 with the anti-VEGF-A antibody B20.4.1 [31]. To directly compare the effects of VEGF-A, Ang-2, and dual inhibition on glioma biology, we tested the effects of these three antibodies vs. control antibody in an identical, clinically relevant dose (5 mg/kg BW every third day) (for treatment groups, see Figure S1A). To follow both morphology and pathophysiological features of glioma cells and tumor blood vessels alike, which would allow deeper insights into the complex world of interactions during the different combination therapies, we established a novel in vivo two-photon microscopy technology. This experimental setup made it possible to determine multiple parameters in the same tumor over multiple time points (Figure S1B).

### 2.2. Differential Vascular Effects of Antiangiogenic Combination Regimens

**Figure 1:**
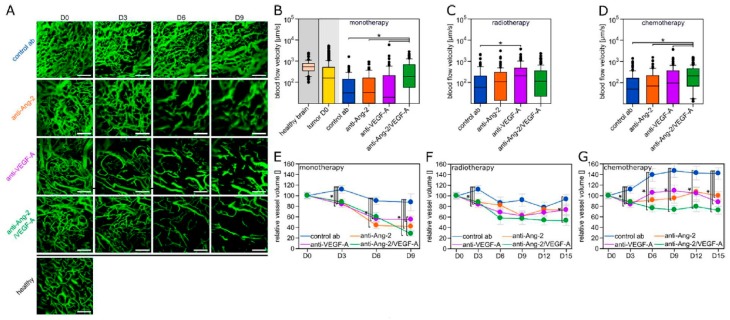
Vascular parameters for antiangiogenic treatment groups in monotherapy, and in combination with radio- or chemotherapy. (**A**) Representative angiograms for control, anti-Ang-2, anti-VEGF-A, and anti-Ang-2/VEGF-A monotherapy in comparison to healthy brain vasculature. Note that morphological vascular normalization occurs preferentially on days three and six under anti-Ang-2/VEGF-A dual inhibition. Scale bars: 150 µm. (**B**–**D**) Microvascular blood flow velocity in the healthy brain, in tumor blood vessels at the beginning of therapy (tumor D0), and on D6 in all four treatment groups. (**B**) Without cytotoxic combination partner; (**C**) in combination with radiotherapy; (**D**) in combination with TMZ chemotherapy. A total of 68–112 vessels from 5–11 animals per group were quantified. Box plots representing median values with 10th, 25th, 75th, and 90th percentiles. * *p* < 0.05 one-way ANOVA on ranks and post hoc Dunn’s test. (**E**–**G**) Vessel volume over time for the different antiangiogenic antibodies given without cytotoxic therapy (**E**) or in combination with radiotherapy (**F**) or chemotherapy (**G**). Overall, 11–23 regions from 6–12 animals per group. Data are expressed as mean ± SD. * *p* < 0.05 one-way ANOVA and post hoc Tukey test.

Dynamic angiograms of the same glioma region over time revealed striking morphological changes indicative of vascular normalization with anti-Ang-2/VEGF-A: blood vessels became thinner, much more ordered, and a clearer hierarchy developed, better resembling blood microvessels of the normal brain (Figure 1A). In contrast, both anti-VEGF-A and anti-Ang-2 monotherapy caused antiangiogenic changes of the tumor vasculature, reducing the number of newly built blood vessels over time when compared to tumors treated with the control antibody, but a clear morphological normalization of the existing tumor vasculature was not evident (Figure 1A). In line with this finding, the microvascular blood flow velocity, a good integrative parameter to measure tumor hemodynamics [32] and a particular robust one to determine functional vascular normalization [7] was decreasing in control tumors over time, while only dual Ang-2/VEGF-A inhibition rescuing levels to those seen in normal brain (Figure 1B). Remarkably, this was different when the antiangiogenic antibodies were combined with radiotherapy, where only anti-VEGF-A achieved a significant normalization of blood flow velocities compared to controls (Figure 1C), while in combination with chemotherapy it was again anti-Ang-2/VEGF-A that stood out as vascular normalization strategy (Figure 1D). Functional vascular normalization was paralleled by morphological vascular normalization in these distinct combination regimens (anti-VEGF-A plus RT, Suppl. Figure 2A; anti-Ang-2/VEGF-A plus chemotherapy, Figure S2B).

As a point of caution, it has been a long matter of debate whether the effects of vascular normalization can actually help the tumor cells gain better access to oxygen and nutrients, thereby creating unwanted effects. Indeed, when quantifying the occurrence of mitotic figures in glioma cells in vivo, we found that antiangiogenic treatments lead to a short-time “burst” of glioma cell proliferation that ceased at later time points and was not present when antiangiogenic agents where combined with chemo- or radiotherapy (Figure S3A–D).

The total microvascular volume was reduced by all three antiangiogenic agents, as monotherapies and in all combinations with chemo- or radiotherapy; consistently, the strongest long-term reductions were seen with anti-Ang-2/VEGF-A (Figure 1E–G; compare also Figure 1A and Figure S2A,B). Of note, this effect was most evident in combination with chemotherapy (Figure 1G).

Together, this data speaks for differential activities of the three antiangiogenic antibodies on important parameters of tumor vascularization and, unexpectedly, for partially divergent effects when they are combined with radio- or chemotherapy.

### 2.3. Tumor Growth Inhibition is Limited to Regimens Where Vascular Normalization Occurs

Tumor growth over time was determined through the cranial window by measuring the area occupied by RFP-positive glioma cells, ensuring that real anti-tumor and not mere anti-edema effects were assessed. Furthermore, we ruled out that anti-edema effects of antiangiogenic therapies influence measurements of gross tumor size by demonstrating that density of tumor cell nuclei in a given tumor volume does not change during all three antiangiogenic treatments (Figure S3E,F).

**Figure 2:**
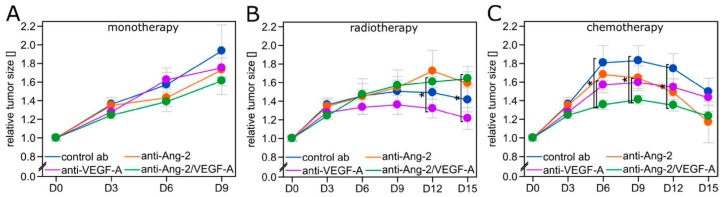
Tumor size over time. Brain tumor size as measured through the cranial window over time in 6–7 animals per group. Antiangiogenic therapy without cytotoxic therapy (**A**) or in combination with radiotherapy (**B**) or temozolomide (TMZ) chemotherapy (**C**). Data are expressed as mean ± SEM. * *p* < 0.05 two-tailed Student’s *t*-test.

All three antiangiogenic therapies did not significantly slow down tumor growth when given without a cytotoxic combination partner (Figure 2A). When combined with radiotherapy, anti-VEGF-A showed strongest tumor growth inhibition, significantly better than with anti-Ang-2/VEGF-A (Figure 2B). This reflects the superiority of this combination regimen regarding vascular normalization (Figure 1C, Figure S1A). In contrast, TMZ chemotherapy was most effective when combined with anti-Ang-2/VEGF-A (Figure 2C), again matching the strongest vascular normalization seen with this combination regimen (Figure 1D, Figure S2B).

### 2.4. Tumor Cell Death Patterns Suggest Improved TMZ Penetration

We have demonstrated before that VEGF pathway inhibition improves the antitumor effects of radiotherapy in glioma due to increased tumor oxygenation during vascular normalization [6]. To get indications whether vascular normalization also helps TMZ chemotherapy by improving tumor penetration of the drug, we analyzed the occurrence of nuclear changes indicative of cell death with respect to blood vessel proximity in all combination regimens in vivo over time. For that we exploited that in addition to cytoplasmatic RFP, glioma cells stably expressed both RFP in the cytoplasm, and GFP in the nucleus.

**Figure 3:**
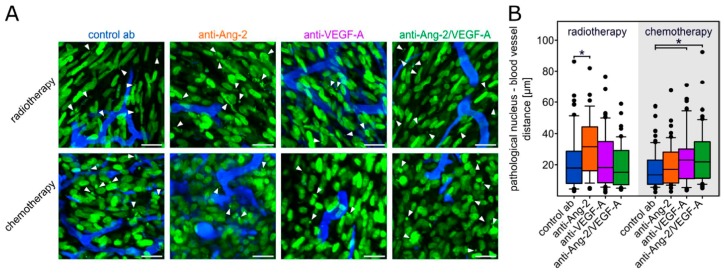
Dynamic changes of tumor cell nuclear parameters. (**A**) Representative images of pathological nuclei in dependence from the vessel distance. Green fluorescent protein (GFP) expressing nuclei are shown in green and the tumor vasculature is visualized in blue. The small arrows are only highlighting typically pathological nuclei. Scale bars: 20 µm. (**B**) Distance of the pathological nuclei from the proximal vessel on D9. A total of 35–68 cells from 4–7 animals per group were quantified. Box plots representing median values with 10th, 25th, 75th, and 90th percentiles. * *p* < 0.05 one-way ANOVA on ranks and post hoc Dunn’s test.

Added to radiotherapy, anti-VEGF-A, the strongest vascular normalization regimen in this combination, did not significantly modify the distance of pathological events in relation to perfused blood vessels (Figure 3A,B). In contrast, when combined with chemotherapy, anti-Ang-2/VEGF-A, but also anti-VEGF-A, did significantly increase the median distance of pathological nuclei to the nearest blood vessel, compared to the control antibody (Figure 3A,B). Together, this data supports the concept that vascular normalization can increase the effectivity of chemotherapy by allowing better drug penetration to the glioma cells.

### 2.5. Tumor Microtube Formation and Cellular Motility Closely Reflect Divergent Responses to Combination Regimens

**Figure 4:**
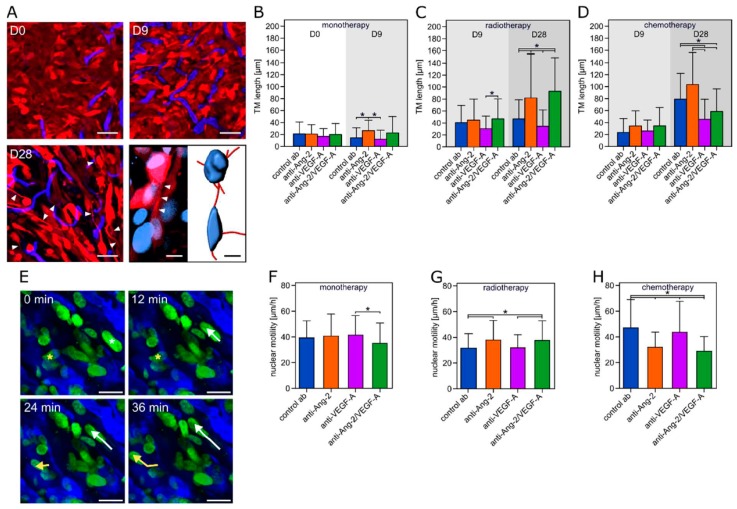
Tumor microtubes (TM) development and tumor cell motility. (**A**) Representative images of cellular morphology including TM development for the control antibody plus TMZ chemotherapy group. Note development of long cellular protrusions of 1–2 µm diameter, which is consistent with the criteria of TMs. Lower right panel: 3D reconstruction of TM-mediated glioma cell connections. Scale bars: 50 µm and 10 µm (right lower corner). (**B**–**D**) TM length for antiangiogenic monotherapy, and combinations with radiotherapy or chemotherapy. *n* = 60 cells from 3 animals per group. (**E**) Representative tracks of the movement of two nuclei over 36 min. Scale bars: 25 µm. (**F**–**H**) Velocity of tumor cell nuclei for the monotherapy and the combined treatment with irradiation or TMZ. *n* = 60–140 nuclei from 3–7 animals per group. Data are expressed as mean ± SD. * *p* < 0.05 one-way ANOVA on ranks and post hoc Dunn’s test.

We have recently discovered that glioma cells extend ultra-long cellular extensions, called tumor microtubes (TMs), to interconnect with each other to a multicellular network in which tumor cells resists the harmful effects of radiotherapy. TMs even increase in response to radiotherapy [3]. Therefore, the occurrence and length of TMs under different therapy strategies was determined on D0, D9, and D28 after the start of the antiangiogenic treatment (Figure 4A–D). In combination with radiotherapy, anti-Ang-2 and anti-Ang-2/VEGF-A both increased TM formation, while anti-VEGF-A (the optimum combination partner) did not. Likewise, in combination with chemotherapy, the ideal combination partner anti-Ang-2/VEGF-A, and also anti-VEGF-A, reduced TM length over time, compared to control and anti-Ang-2 antibodies.

One possible unwanted effect of antiangiogenic therapy is increased tumor cell invasiveness (Figure 4E) [33,34,35,36]. Anti-Ang-2/VEGF-A monotherapy slightly reduced nuclear motility, compared to control and the two other antiangiogenic antibodies (Figure 4F). While anti-Ang-2 and anti-Ang-2/VEGF-A increased motility compared to control when combined with radiotherapy, anti-VEGF-A did not (Figure 4G). In contrast, in combination with chemotherapy, anti-VEGF-A failed to reduce nuclear motility, but anti-Ang-2/VEGF-A and anti-Ang-2 did (Figure 4H).

## 3. Discussion

**Figure 5:**
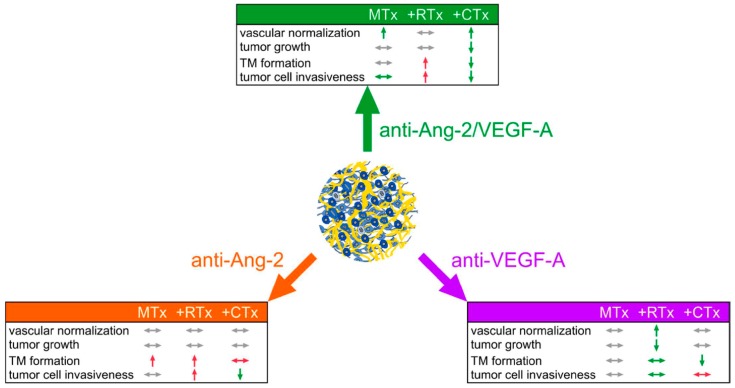
Summary of results. Schematic summary of the different experimental groups: antiangiogenic treatments as monotherapy or in combination with radiotherapy or chemotherapy for the most important parameters. Arrows down: parameter is decreased; arrows up: parameter is increased; sideways arrows: parameter is not affected; green arrows: beneficial effect compared to the other treatment groups; red arrows: unwanted effect compared to the other treatment groups; grey arrows: no effect compared to the other treatment groups.

In this study, we conducted a characterization of different antiangiogenic strategies in combinations with radio- and chemotherapy in glioblastoma. We found that anti-VEGF-A was the optimal combination partner for radiotherapy, while a bispecific antibody inhibiting both Ang-2 and VEGF-A was the best for chemotherapy throughout multiple parameters of tumor progression and therapy resistance. Importantly, there was an excellent correlation with morphological and functional vascular normalization [6,14,18], supporting that this concept has therapeutic relevance for primary brain tumors. Unexpectedly, the cytotoxic combination partner (chemo- vs. radiotherapy) had profound influence on how the antiangiogenic treatments influenced the different parameters of tumor biology, frequently even producing opposite effects (Figure 5).

It has been demonstrated before that the VEGF and angiopoietin pathways are interrelated in glioma, making dual inhibition a plausible strategy. In patients with recurrent GB treated with bevacizumab, plasma Ang-2 concentrations were significantly increased at the time of relapse, pointing towards a potential role of Ang-2 in the development of resistance against VEGF-A targeting treatments [37]. This supports previous preclinical reports that Ang-2 upregulation is typically found during VEGF pathway inhibition [6,9,38]. Furthermore, a particular strong vascular normalization during co-inhibition of the VEGF-A and Ang-2 pathways is also supported by recent preclinical findings [9,11]. Moreover, dose-dependent effects of antiangiogenics need to be taken into account, which can reach from merely normalization effects in lower doses to frank vascular pruning in higher doses, including differential effects on cancer cells [7,14]. The dose selected for this study was a lower dose, which is however still in the range of doses given to patients, were a maximum effect on vascular normalization could be expected, and less vascular regression.

However, dual inhibition of VEGF-A and Ang-2 did only improve the effects of TMZ chemotherapy, which included multiple favorable effects on parameters of glioma progression and therapy resistance. We demonstrate that nuclear changes indicative of glioma cell regression are not limited to the direct perivascular region in this combination anymore, supporting improved tumor penetration of TMZ by anti-Ang-2/VEGF-A co-inhibition. One can wonder why vascular normalization with re-erection of the blood-brain barrier is helping chemotherapy at all. In fact, contradictory findings have been reported whether antiangiogenic therapy improves chemotherapy penetration into the tumor or even hinders it [39,40,41,42,43,44]. However, TMZ is well known to effectively cross the blood-brain barrier (BBB) [45], which is the likely reason why it had similar clinical effects when compared to standard radiotherapy in a recent phase III study of low-grade glioma, where relevant BBB breakdown is normally not present [46]. With such a chemotherapeutic agent, BBB re-erection of any antiangiogenic treatment should be of minor relevance, and better drug delivery to the tumor by increase of its normally low blood flow velocity, but also by decrease of its high interstitial fluid pressure and other pathophysiological tumor features [14] during vascular normalization are likely to improve cytotoxic drug effectiveness. This view is supported by the findings of our study, where anti-Ang-2/VEGF-A was the only antiangiogenic treatment that normalized tumor blood vessels when combined with TMZ chemotherapy, and also significantly reduced tumor growth. Limitations remain. The tumor growth delay detected here might not translate into improved patient survival and the anti-edema effects of antiangiogenics may have clinical benefit even if the tumor load is increasing [47].

VEGF-A is responsible for the stimulation of proliferation and migration of endothelial cells and the enhancement of the vascular permeability [48]. Gliomas and other tumors often show highly elevated expression levels. For this reason, the therapeutic inhibition of this pathway was an important advance and is now targeted for a broad range of cancer entities [26,49]. However, further studies demonstrated that the efficacy of VEGF-A inhibition could be compromised by up-regulation of other angiogenic pathways [50]. One important component of this resistance is Ang-2, which is promoting neovascularization and tumor growth by Tie2 signaling in a VEGF-A independent manner [51,52,53]. While the single inhibition of Ang-2 led to a modest (if at all) beneficial effect on tumor growth and vascular normalization, which is in line with our results, the dual inhibition of both VEGF-A and Ang-2 proved beneficial for the therapy of CNS [9,10,11] and non-CNS [26,29,54,55,56,57,58,59] tumors.

In this study, we provide additional data in which opposite effects of anti-VEGF-A vs. anti-Ang-2 vs. dual inhibition on glioma growth are evident, depending on whether standard chemotherapy or standard radiotherapy is used as the combination partner. This might be best explained by three observations. First, chemotherapy and radiotherapy by themselves (without an active antiangiogenic combination partner) modulate blood vessel morphology, with antiangiogenic, anti-vascular, and partially also vascular normalization effects (control antibody groups: Figure S2, compared to Figure 1A) [60,61,62]. This can explain that the overall change of vascular morphology and function is somewhat unpredictable when an antiangiogenic agent is added. Second, the resulting differential vascular normalization effects observed with the various combination regimens can lead to various levels of tumor hypoxia, which is known to increase resistance to radiotherapy and TMZ [6,14,15,16,17,18]. Lastly, an additional or alternative explanation can be sought in our finding that relevant parameters of tumor resistance (e.g., TM formation, cellular invasiveness) are divergently increased or decreased. It has been described that radiotherapy can increase glioma cell invasiveness [63,64] as one potential mechanism. Importantly, both radio- and chemotherapy can increase the number of TMs and their interconnections and the multicellular networks formed by TMs in gliomas are prime factors of primary and adaptive resistance against radiotherapy and TMZ chemotherapy [3,65,66]. Moreover, TMs drive glioma cell invasion in the brain. An increased glioma cell invasiveness that can occur during inhibition of angiogenesis has been proposed as one major mechanism of resistance against antiangiogenics [33,34,35,36], although this could not be clearly demonstrated in patients yet [67]. Finally, it is very well possible that other well-described effects of antiangiogenics and/or cytotoxic therapies, like modulation of the immune tumor microenvironment and DNA damage response, play a role here. Our study provides the first evidence that antiangiogenic agents can even decrease cellular resistance mechanisms of glioma cells, but only if the right cytotoxic combination partner is selected.

In summary, we provide evidence that inhibition of VEGF-A might be the best combination strategy for radiotherapy, but inhibition of both Ang-2 and VEGF-A for chemotherapy. This provides interesting cues how to best develop dual anti-Ang-2/VEGF-A inhibitors in the clinic: combination with chemotherapy (either adjuvant TMZ in newly diagnosed glioblastoma or lomustine in recurrent glioblastoma) appears the most promising clinical trial strategy, while combination with radiotherapy might even be avoided. Since all phase III studies in primary or recurrent glioblastoma did not find unexpected CNS toxicities or other toxicities when the anti-VEGF-A antibody bevacizumab was combined with chemo-/radiotherapy (Chinot et al. 2014; Gilbert et al. 2014; Wick et al. 2017 [21,22,23]), at least safety and tolerability seem to not be a major issue with these treatment strategies. The most important consequence of the surprisingly complex interactions between antiangiogenic and cytotoxic treatments reported here is to better study them in preclinical and early clinical settings in the future to avoid testing uninformed combination regimens in controlled clinical trials.

## 4. Materials and Methods

### 4.1. Cell Culture

Cell culture was done under adherent conditions with Dulbeccos’s Modified Eagle’s Medium (DMEM, Sigma-Aldrich, Munich, Germany), which was supplemented with 10% fetal bovine serum (FBS, Sigma-Aldrich, Munich, Germany) and 1% penicillin-streptomycin (PS, Sigma-Aldrich, Munich, Germany). For the cultured cells a contamination and authentification test was done by Multiplexion (Heidelberg, Germany). U-87MG, a human glioblastoma cell line obtained from the American Type Culture Collection (LGC Standards, Wesel, Germany), which is growing angiogenic in the mouse brain was used. O^6^-methylguanine-DNA methyltransferase (MGMT) promotor methylation of this cell line was already confirmed in former publications [68]. Furthermore, when 24 brain tumor cell lines were analyzed for VEGF-A and Ang-2 mRNA expression, the U-87MG cell line showed both, being considerably representative with respect to expression levels. The cells were stably co-transduced with the red cytoplasmic construct LeGO-T2 (plasmid #27342 Addgene, Cambridge, MA, USA) and the green nuclear fluorescent plasmid LV-GFP (plasmid #25999 Addgene, Cambridge, MA, USA). The co-transduced cells were selected by fluorescence-associated cell sorting (FACSAria^TM^ Special Order System, BD Biosciences, Heidelberg, Germany). To distinguish between single and double transduced cells, compensation was used. To separate dead and alive cells, propidium iodide (Sigma-Aldrich, Munich, Germany) staining was used.

### 4.2. Animals and Surgical Procedures

Naval Medical Research Institute (NMRI) nude male mice, between 8 and 10 weeks old (Charles River, Sulzfeld, Germany) were used to study the angiogenesis of human brain tumor cells within the mouse brain. All efforts were made to minimize animal suffering and to reduce the number of animals used. The operation of the chronic cranial window was done as previously described [3,69]. One week after window implantation a 1 µL cell suspension, containing 50,000 tumor cells, was injected cortically 500 µm deep with a stereotactical injector (Hamilton, Bonaduz, Switzerland and Stoelting, Wood Dale, IL, USA). When the tumor reached a mean diameter of 2 mm in vivo imaging and therapy was started. The animals were sacrificed when they got moribund and/or developed a weight loss of over 20%, which was particularly important in light of the frequent intravital imaging sessions that were an additional burden for tumor-bearing mice. The animals were treated every third day with the control (MOPC21), the anti-Ang-2 (LC06, RO6872894), the anti-VEGF-A (B20.4.1, RO6872895), or the anti-Ang-2/VEGF-A antibodies (LC06/B20.4.1, RO6872840) (F. Hoffmann-La Roche, Penzberg, Germany). All antibodies were administered in the same concentration of 5 mg/kg body weight (bw). For the radiotherapy group, tumors were irradiated with 7 Gy on D4, D5, and D6 (three consecutive days; total dose 21 Gy) after the start of the antiangiogenic treatment. The radiation was done with a 6 MV linear accelerator with a 6 mm collimator (adjusted to the window size) at a dose rate of 3 Gy min^−1^ (Artiste, Siemens, Erlangen, Germany). The administered radiation dose is in the range of the commonly used 60 Gy in 2 Gy fractions for glioma patients, assuming an α/β of ~10 in the linear quadratic model, and taking into account a radiation time of three days (Osswald et al. 2015; Winkler et al. 2004 [3,6]). For the chemotherapy group, animals were orally administered with TMZ (Schering-Plough, Kenilworth, NJ, USA) using a feeding needle on the three consecutive days during D4–D6 of therapy. TMZ concentration was 20 mg/kg bw per day (Figure S3A,B). This dose was selected because it was shown to exert measurable anti-tumor effects as single agent in preliminary studies on U87 and other glioma models (Weil et al. 2017 [65]) in our laboratory, but still low enough to be comparable to bioavailable doses given to patients. All animal procedures were performed in accordance with the institutional laboratory animal research guidelines after approval of the Regierungspräsidium Karlsruhe, Germany (governmental authority).

### 4.3. In Vivo Multiphoton Laser Scanning Microscopy (MPLSM)

In vivo imaging was performed with a LSM 7MP microscope (Carl Zeiss Microscopy, Jena, Germany) provided with a Coherent Chameleon UltraII laser (Coherent, Glasgow, UK) with a 500–550 nm and a 575–610 nm band pass filter. With the following wavelengths fluorophores were detected: 750  nm (FITC-dextrane, tdTomato) and 850 nm (GFP, TRITC-dextrane). To prevent phototoxic effects laser power was always kept as low as possible. During the imaging process animals were anaesthetized with a low gas narcosis including 1.5% isoflurane (Baxter, Unterschleißheim, Germany) and 98.5% oxygen (Guttroff, Heidelberg, Germany). During imaging body temperature was kept constantly at 37 °C by a heating pad. To acquire angiographies of brain blood vessel, 0.1 mL high molecular dextrans were injected intravenously: tetramethylrhodamine isothiocyanate (TRITC, 500 kDa, 10 mg mL^−1^, Sigma-Aldrich, Munich, Germany), and fluorescein isothiocyanate (FITC, 2 MDa, 10 mg mL^−1^, Sigma-Aldrich, Munich, Germany). The angiographic image (size: 607.28 × 607.28 × 123 µm) allowed longitudinal tracking of two consecutive regions in the center of the brain tumor, which was done every third day from D0 to D15 after beginning with antiangiogenic treatment.

The large cranial window also allowed to study the entire tumor up to a depth of up to 1000 µm that sufficiently allowed to assess the tumor diameter. Verification experiments were performed, comparing the diameter measured by in vivo two-photon microscopy with the maximum diameter measured by standard histology and fluorescence microscopy of tumor-bearing brain sections. Here, a strong correlation was detected [7]. The mean tumor diameter under the cranial window was measured by a tile scan in a depth of 400 µm.

On D0 and D6, microvascular blood flow velocity was measured by a line scan as described before [7]. The cellular morphology and the length of the TMs were determined in a depth of 51 µm on D0, D9, and D28. On D3, D9, and D15 the nuclei were detected with a higher magnification (size: 151.82 × 151.82 × 48 µm) to quantify the nuclear density and the nuclear morphology. Additionally, the nuclear motility of the cells of the same region (size: 151.82 × 151.82 × 99 µm) was measured over 1.5 h.

### 4.4. Quantification and Visualization of MPLSM Data

In vivo images were recorded with the ZEN Software (Carl Zeiss Microscopy, Jena, Germany). Images were analyzed with the ImageJ 1.51f software (Wayne Rasband, National Institutes of Health, Bethesda, MD, USA). The diameter was determined by a tile scan in a depth of about 400 µm, where the tumor bulk reached its major dimensions. The diameter was calculated as the average of the minor and major axis of an ellipse. For quantification the diameters were normalized to the mean diameter on D0.

The blood flow velocity was measured by a line scan with a minimum length of 10 µm, detecting 2000 events in microvessels. Moreover, 16 randomly chosen vessels were measured per animal. The resulting scan identifies single erythrocytes as angular black lines, where the x-axis is according to the length of the detected distance and the y-axis is the elapsed time of the measurement. The angle is converging more and more to 90° when the cells are static. By knowing the resolution of both parameters and by the measurement of the slope of 30 randomly chosen red blood cells, a calculation of the mean velocity is possible, by inversion of the result.

For the nuclear density and the quantification of mitotic cells, stacks cropped to thickness of 67 µm (151.82 × 151.82 µm) in a depth of 35–102 µm were analyzed. The nuclear density was calculated by dividing the manually counted cell number with the volume of the stack. For the quantification of the mitotic activity, the percentage of mitotic cells was detected. Due to distances up to 100 µm between vessels and pathological nuclei, the whole and not the cropped stacks were analyzed manually. In control animals, nuclear size and shape was very homogenous. In distinct treatment groups, nuclear morphological changes of apoptosis or necrosis where evident. Tortuous, abnormally flexed, strongly condensed or swollen nuclei were evaluated as pathological (see Figure 3A). The distance between the center of the pathological nucleus and the exterior of the wall of the blood vessels was measured manually.

TMs were identified as thin and long cytoplasmatic protrusions, which often interconnect tumor cells [3]. For the measurement of the TM length single slices in a depth of 51 µm were quantified for two regions per animal. For every region 30 random cells were picked, where the length of the longest and most prominent TM of the cell was determined.

Cellular invasiveness was detected for 3D stacks in a depth of 51–150 µm, which were recorded in 40 cycles long (over 1.5 h lasting) time series. For 20 randomly chosen nuclei, x, y, and z coordinates were identified for 10 different time points. Since the expansion of the tissue by laser irradiation and breathing artefacts also modifies coordinates, striking blood vessel bifurcations were used as an inertial system.

3D representative pictures for the angiograms, nuclear morphologies, nuclear densities, nuclear motilities and the 3D rendering of the TMs were acquired with Imaris 7.5.1 (Bitplane, Zürich, Switzerland). Finally, the pictures were edited with Inkscape 0.91 (GNU General Public License) and GIMP 2.8.14 (GNU General Public License).

### 4.5. Statistical Analysis

The measured results were arranged in Excel 2016 (Microsoft Corporation, Redmond, WA, USA) and tested for outliers by a Nalimov test. The outlier-cleared datasets were transferred to the statistic software SigmaPlot 13.0 (Systat Software, San Jose, CA, USA). Equal variance and normal distribution were tested (Kolmogorov-Smirnov or Shapiro-Wilk). For results with normal distribution and equal variance, the two-sided Student’s *t*-test was used. Otherwise, the Mann-Whitney U statistic was applied. For measurements with more than two groups, ANOVA on ranks or ANOVA was used in combination with the suitable post-hoc test (Dunn’s method for the non-parametric and the Tukey test for the parametric). Results were significant from the corresponding control at a critical p-value below 0.05. Results were plotted in a line diagram by presenting the mean ± standard error of mean (SEM). In a bar chart the results were displayed as mean ± standard deviation (SD). Box plots depicted the median values with the percentiles and error bars. Results shown in a line diagram and in a bar chart have a linear scale; results in a box plot had a common logarithm scale for the ordinate. Finally, the pictures were finished with the graphic editors Inkscape 0.91 (GNU General Public License) and GIMP 2.8.14 (GNU General Public License).

## 5. Conclusions

Despite very high angiogenic activity of malignant gliomas, the current clinical effectiveness of antiangiogenic drugs and their combination regimens falls short of expectations. Here we show that inhibition of VEGF-A might be the best combination strategy for radiotherapy, but dual inhibition of Ang-2 plus VEGF-A for chemotherapy. This provides interesting cues how to best develop dual anti-Ang-2/VEGF-A inhibitors in the clinic. Combination with chemotherapy (either with adjuvant TMZ in primary glioblastoma, or lomustine in recurrent glioblastoma) appears most promising. Remarkably, the VEGF-A blocking antibody bevacizumab has never been tested with radiotherapy alone in a controlled clinical trial, which might be its optimal combination partner according to the results of this study. The most important consequence of the surprisingly complex interactions between antiangiogenic and cytotoxic treatments is to better study them in preclinical and early clinical settings in the future - to avoid testing uninformed combination regimens in controlled clinical trials.

## Supplementary Materials

The following are available online at https://www.mdpi.com/2072-6694/11/3/314/s1, Figure S1: Experimental design, Figure S2: Differential changes of tumor blood vessel angiograms during combination of antiangiogenic treatment with radio- or chemotherapy, Figure S3: Transient pro-mitotic effects, and nuclear densities.
