# Supplementary material for: Differential Effects of Ang-2/VEGF-A Inhibiting Antibodies in Combination with Radio- or Chemotherapy in Glioma
Source: Cancers (Basel). 2019 Mar 6;11(3):314. doi: 10.3390/cancers11030314 (PMC6468722; doi:10.3390/cancers11030314)
Supplement: Supplementary file 1 [file cancers-11-00314-s001.pdf]

cancers

MDPI

Supplementary Materials: Differential effects of Ang-2/VEGF-A inhibiting antibodies in combination with radio- or chemotherapy in glioma

Gergely Solecki, Matthias Osswald, Daniel Weber, Malte Glock, Miriam Ratliff, Hans-Joachim Müller, Oliver Krieter, Yvonne Kienast, Wolfgang Wick and Frank Winkler

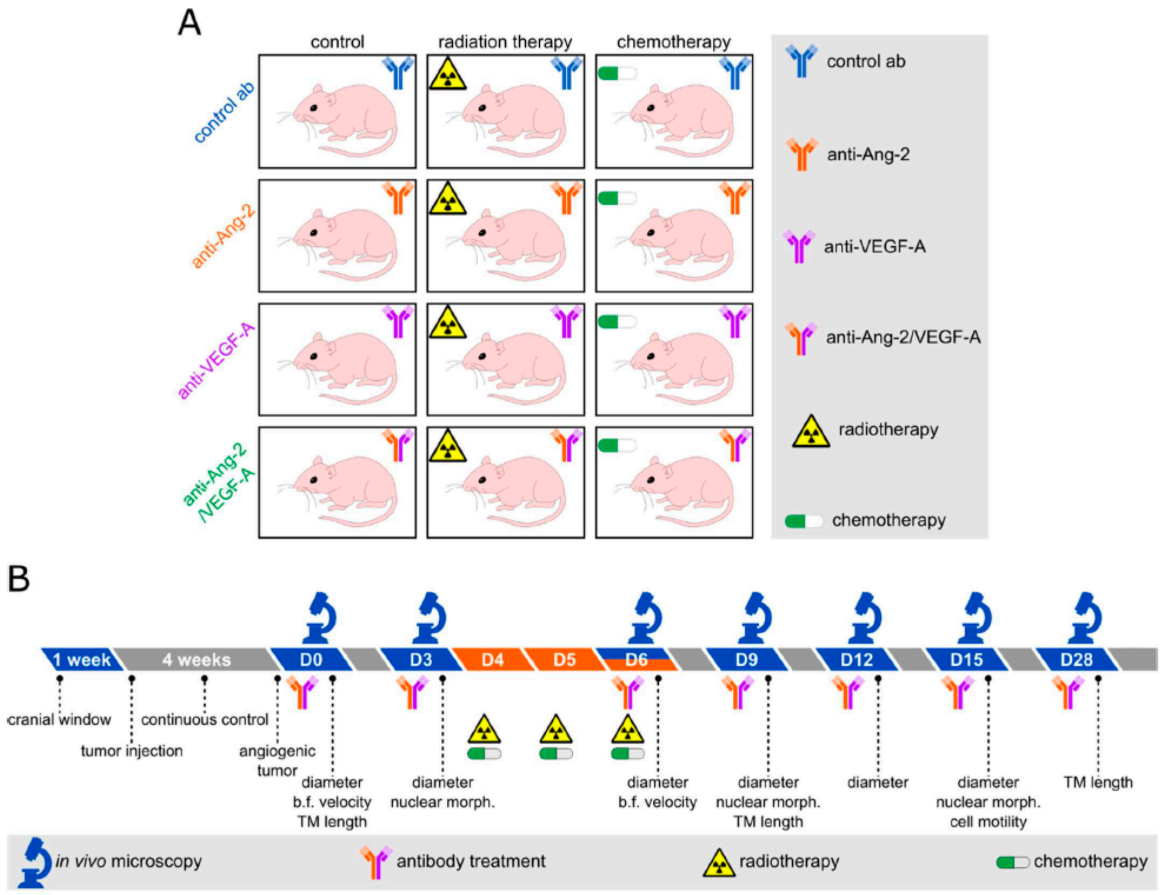

Figure S1. Experimental design. (**A**) Schematic diagram of the different experimental groups: antiangiogenic treatments as monotherapy, or in combination with radiotherapy or chemotherapy. (**B**) Time schedule of the experimental procedures.

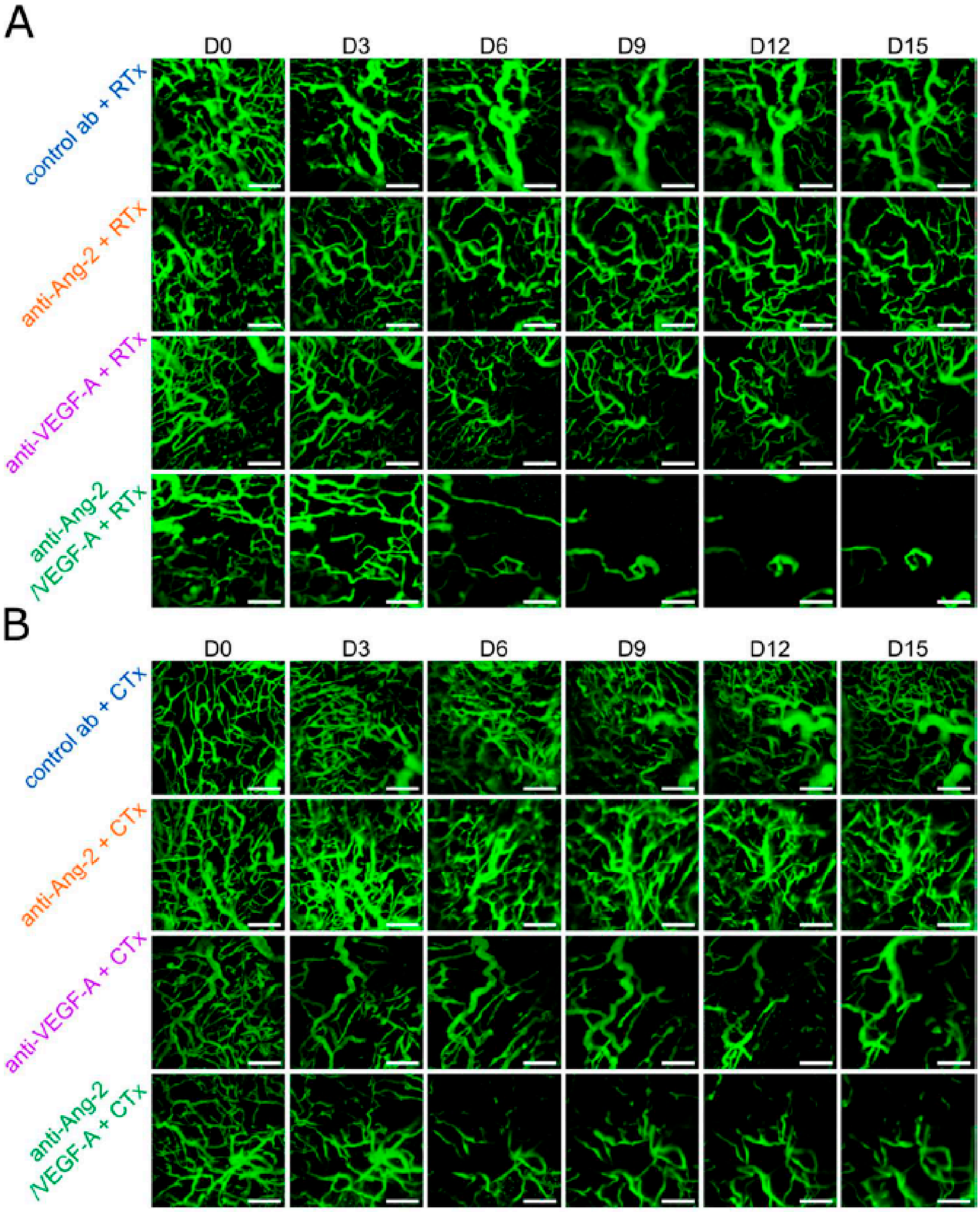

Figure S2. Differential changes of tumor blood vessel angiograms during combination of antiangiogenic treatment with radio- or chemotherapy. Representative images over time. Note the superior vascular normalization (reduction of diameter of enlarged microvessels; reestablishment of more normal hierarchical structure of the vasculature, decrease of aberrant loops and shortcuts) when anti-VEGF-A is combined with radiotherapy (**A**), and anti-Ang-2/VEGF-A with chemotherapy (**B**). Scale bars: 150 µm.

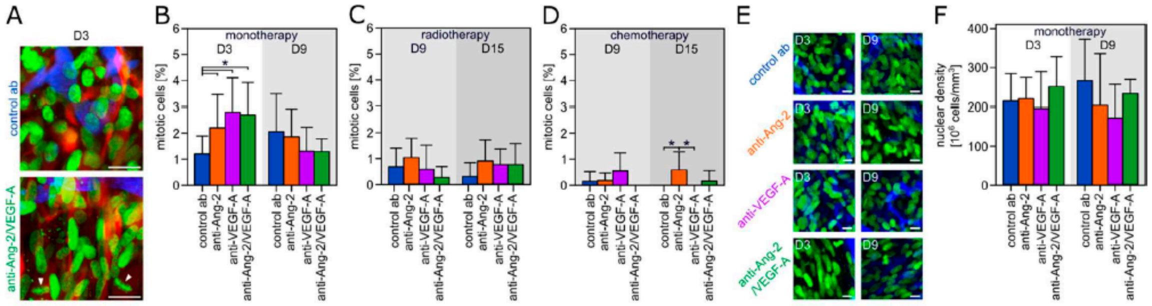

Figure S3. Transient pro-mitotic effects, and nuclear densities. (**A**) Representative images of mitotic activity for the control and anti-Ang-2/VEGF-A antibody. Scale bars: 20 μm. (**B**–**D**) Fraction of mitotic cells during antiangiogenic monotherapy and in combination with radio- or chemotherapy. 5–8 animals per group. Data are expressed as mean ± SD. * $p < 0.05$ one-way ANOVA and post hoc Dunn's test. (**E**) Representative images of the nuclear densities for different antiangiogenic treatment modalities. Scale bars: 25 μm. (**F**) Nuclear density per tumor volume during antiangiogenic monotherapy. 5–8 animals per group. Data are expressed as mean ± SD. * $p < 0.05$ one-way ANOVA and post hoc Dunn's test.

© 2019 by the authors. Licensee MDPI, Basel, Switzerland. This article is an open access article distributed under the terms and conditions of the Creative Commons Attribution (CC BY) license (http://creativecommons.org/licenses/by/4.0/).
